# Genome-wide association study of water-use efficiency and shoot biomass conferred by *V. berlandieri* rootstocks in grapevine

**DOI:** 10.1186/s12870-026-08542-6

**Published:** 2026-03-27

**Authors:** Louis Blois, Marina de Miguel, Joachim Schmid, Elisa Marguerit

**Affiliations:** 1 EGFV, Univ. Bordeaux, Bordeaux Sciences Agro, INRAE, ISVV, Villenave d’Ornon, F-33882 France; 2 Department of Grapevine Breeding, Geisenheim University, Von Lade Str. 1, Geisenheim, 65366 Germany

**Keywords:** GWAS, Carbon isotope discrimination, Vegetative growth, Root system, Perennial, Rootstock

## Abstract

**Background:**

Deciphering the genetic basis of above and below ground organs communication is essential for plant adaptation. In grafted perennial crops, such as grapevine, identifying genes from the roots system controlling the expression of shoot traits is essential to improve plant adaptation to environmental conditions through rootstock breeding. However, the grapevine rootstocks genetic control on conferred scion traits has rarely been explored and even less considering genetic diversity at the intra-species level through GWAS.

In this study we used a *Vitis berlandieri* natural population comprising 211 genotypes, grafted with a single scion genotype, to identify genetic regions associated with water use efficiency and shoot biomass conferred to the scion using GWAS in field conditions. Water use efficiency was evaluated by δ^13^C over three years and the shoot biomass produced every year during the first two years of growth after plantation.

**Results:**

δ^13^C and shoot biomass were not correlated. δ^13^C indicated a moderate broad sense heritability from 0.34 to 0.46 in the absence or under moderate water deficit. Heritability falls to zero under strong water deficit indicating a major contribution of environmental conditions. The shoot biomass heritability was moderate to strong from 0.34 to 0.70. One QTL for δ^13^C explained 54% of the genetic variance. The QTL was associated with a gene homolog to *AT4G22790*, coding for a mate family protein involved in stomatal aperture regulation in response to carbon dioxide concentration. Four QTL for shoot biomass were linked with genes homologous to AT5G19350, AT4G37130, AT1G22020, and AT1G15780 known to be involved in vegetative growth and root development, hormonal signalling, photorespiration, and response to light, respectively.

**Conclusion:**

This study represents the first GWAS carried out for scion traits conferred by a natural *Vitis berlandieri* rootstock population in a field experiment. It contributes to the understanding of the genetic basis of shoot plant performance regulated by roots. Ultimately, these outcomes provide valuable targets for breeding programs suggesting that rootstocks selection based on genetic information and morphological traits could improve crop adaptation to future environments, contributing to sustainable agriculture.

**Supplementary Information:**

The online version contains supplementary material available at 10.1186/s12870-026-08542-6.

## Background

Climate change has placed plant adaptation at the forefront of concerns. Deciphering the genetic basis of key traits related to plant performance is crucial to improve crop adaptation through the selection of plant material [1]. One of the most ancient techniques to improve crop production and increase tolerance to biotic and abiotic stress is grafting. In grafted plants, adaptation can be achieved through breeding of scion and/or rootstock. Beyond its use in horticultural production, grafting is reputed as an important research tool, because it allows to decipher signalling mechanisms related to root–shoot communication. Through this technique, there have been significant breakthroughs about the underlying mechanisms involved in the regulation of developmental and stress responses at the whole plant level [2]. The rootstock provides the root system of the plant and manages the absorption of water and nutrients. It therefore plays an essential role in regulating the growth of the entire plant and its tolerance to abiotic stresses, such as water deficit [3]. Rootstocks can be bred to modify scion biomass, earliness, productivity, fruit quality [4], and tolerance to biotic and abiotic stresses [5]. However, the underlying mechanisms remain unclear [5].

Root system has been explored in annual crops as a means of improving plant productivity or water-deficit tolerance [6, 7]. However, the characterization of root systems in perennials involves additional challenges related to the long-term development, because of the diverse range of environmental biotic and abiotic pressures faced by the plant during its lifetime. Thus, in perennial crops, root-related traits associated with water deficit tolerance or plant growth need more investigations. Conducting experiments with grafted plants where only one scion genotype is combined with a panel of diversity of rootstocks (i.e. the genetic variability comes from the rootstock) is an ideal strategy to unravel the influence of the root compartment on the traits of interest at the above-ground level. To our knowledge, such experiments, allowing to implement quantitative genetic approaches in perennial plants have never been conducted in non-controlled conditions.

Grapevine is the most important grafted horticultural crop. In grapevine, scions are derived from the species *Vitis vinifera,* which has been the focus of plant breeding over hundreds of years [8]. Grapevine rootstocks mostly originate from crosses between American *Vitis* species (*Vitis rupestris*, *Vitis riparia*, and *Vitis berlandieri*), each genetic background covering a large genetic diversity in nature [9–12]. However, there has been little research on American *Vitis* species obtained from natural areas and no research was carried out in field conditions for agronomical traits of interest. In grapevine, the rootstock has been shown to contribute to shoot biomass variability [13]. In addition, scion biomass is correlated with fruit quality and wine sensory properties [14], yield [15], and plant water use efficiency (WUE) [16].

The increasing frequency and severity of water deficit events are a major concern in the context of climate change for viticulture. WUE can impact plant survival, growth, and reproduction, especially under water limitation. Plants with higher WUE lose less water per unit of carbon gained and this allows the maintenance of metabolic activity when water availability is low. In grapevine, grafting allowed to increase WUE for the Sultana variety compared to own-rooted plants [17]. Several studies have shown that rootstock genotype affects grape scion responses to water deficit, suggesting the presence of genetic determinants at the rootstock level [18–24]. In a grape progeny issued from a cross *V. riparia* × Cabernet-Sauvignon and used as rootstocks [25], quantitative trait loci (QTL) were identified for δ^13^C measured at the scion level on the linkage groups 12 and 3 and explained from 2.7% to 13.8% of the phenotypic variance over three years. A genome wide association study (GWAS) for water deficit responses in grapevine was carried out in a panel of 279 cultivars of *Vitis vinifera* L [26]. In addition, eight QTL for δ^13^C in field conditions [27] in a non-vinifera *Vitis* association population were identified and 24 markers associated with transpiration-related traits were detected. However, this study used a panel of ungrafted accessions issued from a germplasm collection which failed to explore the rootstocks intra species genetic diversity. Recently, the exploration of the genetic diversity of *V. berlandieri,* an American wild *Vitis* species, has shown a genetic structure associated with environmental conditions of their natural habitats [12] highlighting potential subgroups adaptation to specific environmental conditions and valuable genetic resources for grape rootstocks breeding. *Vitis berlandieri* is an endemic species from the dry and chalky area of the Edward plateau in Texas, USA [28]. Few genotypes have been used in grapevine rootstocks hybridization for the limestone and water deficit tolerances they can confer to their progeny [29]. *Vitis berlandieri* has been considered as a reference genetic background in drought tolerance studies in grapevine [18–20, 30] but has not been explored deeper for its genetic diversity and the genetic determinants conferring such tolerance remain unknown.

The aims of this work were: i) to estimate the extent of genetic variability of above-ground WUE and biomass regulated by roots; ii) to unravel the genetic basis of above-ground WUE and biomass regulated by roots; iii) to link the genetic variation of root traits with scion performance in field conditions using grapevine as model species.

## Methods

### Origin of the plant material and field conditions

The rootstock plant material consisted of half-sibs derived from 78 female plants (open-pollinated families) of wild *V. berlandieri* previously described in [12]. To summarize, seeds were sampled from 78 female plants in natural conditions and were used to create a large population of 5,000 genotypes planted in field at the Grapevine Breeding Department of Geisenheim University, Germany [28]. In this population, 286 genotypes were identified with the aim to sample four to five genotypes per initial female plant. It allowed to conserve the genetic structure of female plants leading to a relatedness structure in the population.

Cuttings of the selected 286 genotypes were used as rootstocks and grafted with Riesling (clone 24–209) scions in 2019 and 2020 at the Grapevine Breeding Department of the Geisenheim University, Germany, with the aim to obtain 5 graft replicates per *V. berlandieri* genotype. Cuttings of both graft partners were sampled in the field as 20 cm-long dormant wood pieces in February. Omega grafting was done in March trying to match the scion and rootstocks diameters. After one month in a warm room, a thumb test was done on each plant by applying a moderate pressure on the scion to check that it is firmly attached to the rootstock. The plants that failed the thumb test were eliminated. Then, plants were grown in plastic containers for one month. After this period, plants were sorted again for their vegetative and root development to evaluate graft quality allowing the elimination of any plant with defective connection between the two partners. In total, 211 genotypes belonging to 72 over the 78 initially sampled female plants had successfully grafted plants. They were grown in individual pots for one year in a greenhouse with no water limiting condition and protection products were used against *Plasmopara viticola* and *Erysiphe necator*. After one year, these plants were then sent and planted in the vineyard of the *Unité expérimentale Vigne et Vin Bordeaux Grande Ferrade Villenave d’Ornon*, in France (N 44° 47′ 32.928″ O 0° 34′ 44.468″).

### Genetic composition and field management of the population

As previously mentioned, due to the variable grafting success, the studied population represented 72 female plants and was composed by one to five genotypes per initial female plants with an average of 2.9. In total, 211 V*. berlandieri* genotypes were represented by one to five replicates.

The field experimental design consisted of seven blocks (Additional Fig. 1). Blocks one to five were planted in 2020 with the plants grafted in 2019 (Experiment 1, consisting in six rows). Blocks six and seven were planted in 2021 with the plants grafted in 2020 (Experiment 2, consisting in four rows). In total, 823 plants were planted (183 genotypes, resulting in 519 plants in Experiment 1 and 142 genotypes, resulting in 304 plants in Experiment 2). The variable grafting success led to an unbalanced and incomplete experimental design (see Additional Table 1) which were accounted for using appropriate statistical approaches described below.

In addition to the *V. berlandieri* genotypes, commercial rootstocks were included and distributed across the field plot. The commercial rootstocks were 110R, SO4, Börner (present in both experiments) and 5BB (included only in Experiment 2), each represented by five replicates per experiment. Commercial rootstocks followed the same grafting procedure as the *V. berlandieri* population at the same periods.

In the field, plants were grown with an inter-row distance of 1.6 m and an inter-plant distance of 0.85 m and only one shoot for the first two years. Weed growth between rows and within the row of vines was limited by mowing. The field was rainfed and local climatic conditions (rainfall, maximum temperature, minimum temperature, mean temperature, and radiation) were recorded hourly and averaged monthly with an automated weather station (Additional Table 2).

### Water-use efficiency

Plant WUE was evaluated four times by determining leaf δ^13^C levels, in 2020, 2021, and twice in 2022 (2022a and 2022b). The number of leaves was counted every two or three weeks from the start of June until the end of August. The number of leaves corresponded to the total number of leaves on the single shoot retained, including the partially expanded leaves. By comparing changes in the number of leaves and the climatic conditions experienced by plants during the summer, it was possible to select a leaf that had developed during the same period on each plant. The leaf selected was dried in an oven at 80 °C for 2 weeks. The dried leaves were ground individually with a Retsch MM 400 mixer mill and 1 ± 0.05 mg of the resulting powder was placed in a pressed tin capsule (6 × 4 mm).

Carbon isotope composition (δ^13^C) was analysed at the INRAE Silvatech platform (Nancy, France) through an elemental analyser coupled with an isotope ratio mass spectrometer (Isoprime100, Elementar). The δ ^13^C value is expressed in ‰ relative to the isotope ratio of the Vienna Pee Dee Belemnite (VPDB) standard [31]:

1 $${\delta }^{13}C \left(0/100 \right)= \left[\frac{{R}_{sample}}{{R}_{std}}-1\right]\times 1000$$

where R_sample_ and R_std_ are the ^13^C:^12^C ratio of the sample and the standard, respectively. Pre-dawn leaf water potential (ψ_pd_) was measured several times in the field over the summer. Measurements were performed with a Scholander-type pressure chamber on different genotypes (n = 18 to 64) spread over the experimental plots to determine the overall water status of the plants in the field.

### Scion annual biomass

The impact of the rootstock on scion annual biomass was evaluated by determining pruning weight (PW) at the end of the growing period. PW is the weight of the shoot produced by the plant during the course of one year. It was measured at the end of the first two growing seasons for each experiment while the plants were grown with a single shoot from which lateral shoots were removed. Each single shoot was pruned and fresh weight was measured directly in field during one single day. During the winter pruning period, all shoots were woody and leafless. During pruning, two buds were left on each plant to ensure the vegetative regrowth the following year. If possible, the shoot from the more distal bud was retained. In case of bud injury or shoot breakage during the first weeks of growth, the shoot from the basal bud was retained for the growing season. Annual biomass was determined on March 17, 2021, April 1, 2022, and April 4, 2023. The measurements obtained corresponded to the shoot plant biomass produced in 2020, 2021, and 2022, respectively. Annual biomass was not measured for 2023 in Experiment 1 because the growth offset between genotypes led to irregular pruning methods. It made it impossible to apply the same management practices to the vegetative parts, introducing evident bias in the genotype performances for the annual shoot biomass production.

### Statistical analysis

The commercial rootstocks were not considered in the statistical analysis because they could artificially reduce or increase the phenotypic and genetic variance of the *V. berlandieri* population. Thus, the best linear unbiased predictors (BLUP) and the best linear unbiased estimates (BLUE) presented hereafter are only estimated on the *V. berlandieri* population in order to avoid bias related to the variability of distinct genetic groups from commercial rootstocks.

BLUPs were estimated for each trait with the following model (Eq. 2), which gave the best fit of residuals and for which no collinearity between covariables was observed (based on the Bayesian information criterion, BIC):

2 $${P}_{gik}=\mu +{G}_{g}+{B}_{i}+{Y}_{k}+{\varepsilon }_{gik}$$

where *P*_gik_ is the phenotypic value of the *g*^*th*^ genotype (*G*_*g*_) in the *i*^th^ block (*B*_*i*_), for the *k*^*t*h^ year of measurement (*Y*_k_) and ε_gik is_ the residual variance. Genotype *G* and the block *B* were considered as random effects in the model, to obtain a variance–covariance matrix for the calculation of broad-sense heritability (H^2^). The year effect was considered as a fixed effect. There were no common blocks to the two years of planting. As a result, the “experiment” effect was collinear with the block effect and was therefore not included in the model. Biomass followed an exponential-like distribution. We therefore used the square roots of phenotypic values and allowed the residual distribution to get close to normality.

The broad-sense heritability of traits was calculated as follows (Eq. 3):

3 $${H}^{2}= \frac{\sigma g^2}{\sigma g^2 + (\sigma e^2/nrep)}$$

where *H*^*2*^ is the broad-sense heritability of the trait, $\sigma g^2$ is the variance explained by the genotype effect and *σ* $e^2/nrep$ is the residual variance extracted from the model divided by the mean number of repetitions per genotype in the population [32]. These models were computed with R software (R version 3.6.1), using the lme4 package [33].

Relationships between aerial traits were tested using Pearson correlation analysis. The R^2^ was the square of the Pearson correlation coefficient. Correlations between the scion phenotype and root-related traits measured on the same plants at a juvenile stage (reported in Blois et al., 12) were explored. The root traits considered were the average root diameter, the root dry weight, the total root diameter, the total root number and the number and proportion of small [0, 1) mm, medium [1, 2) mm, and large roots [2, ∞) mm. The BLUE values obtained for each trait (described in Eq. (4), below) were used to calculate Pearson correlations between the genetic values of the traits. Allelic group for genetic variants selected in GWAS based on BLUE values were compared using ANOVA based on their phenotypic values for each year of measurement. The ANOVA analysis were followed by Tukey’s honestly significant difference (HSD) test.Graphics were constructed with the ggplot2 package in R software [34].

### Genome-wide association study

For assessment of the estimated genetic values of genotypes for δ^13^C and biomass without variance shrinkage due to predictive models. More precisely, the genetic value of genotypes supported by few replicates is strongly pulled toward the population mean reducing the trait variability in the population using BLUP. BLUEs were calculated with the following model, with the genotype considered as fixed effects compared to [2]. The intercepts of each genotype were then used as the new phenotypic value in GWAS.

4 $${P}_{ghi}=\mu +{G}_{g}+{Y}_{h}+{B}_{i}+{\varepsilon }_{ghi}$$

where µ is the mean value of the trait in the population, G_g_ is the effect of the *g*^*th*^ genotype, *Y*_*h*_ is the effect of the *h*^*th*^ year of measurement and *B*_*i*_ is the effect of the *i*^*th*^ block.

According to this procedure, we used only one BLUE value per genotype for the two years of measurement and the two experiments. The estimation of BLUP and BLUE with models including both experiments increased the statistical power of our analysis by increasing sample size. The mean number of repetitions by genotype also increased from 2.5 to 6.2.

The set of single nucleotide polymorphism (SNP) was obtained with a genotyping by sequencing method described previously [12]. We performed the GWAS with the Bayesian information and linkage disequilibrium iteratively nested keyway (BLINK) model, which has the best performance for the detection of significant markers [35]. The BLINK model was used in the genome association and prediction integrated tool (GAPIT3) [36] with default settings, implementation by major allele and MAF > 0.05 filtration. With these specifications, 87,589 SNPs were retained for analyses according to a previously described protocol [37]. Population structure was considered as a covariate with K = 2 [12]. Kinship was derived from pseudo-QTN information, directly in BLINK. Bonferroni correction was used to calculate the significance thresholds, which were set at 0.05/*n* and 0.01/*n*, where “*n*” is the number of markers used. The variance explained by significant SNPs was estimated from BLINK results in GAPIT with a mixed linear model. GWAS was not performed with the 2022 measurement for δ^13^C due to the increasing of water deficit which reduced the genetic variance of the trait challenging the correction of the environmental effect (see results section). For GWAS, genes associated with significant markers were extracted from the *V. berlandieri* genome within a window corresponding to the extent of LD, defined by the physical distance at which r^2^ = 0.2, as outlined in [38] on the relevant chromosome. The mean decay of linkage disequilibrium was observed to be 2.2 kb as reported by [12].

This procedure identified the genes linked to all the significant markers. Gene functions were defined according to the information available from UniProt [39] and Tair [40].

## Results

### The low to moderate heritability for water-use efficiency depended on water deficit intensity

**Fig. 1:**
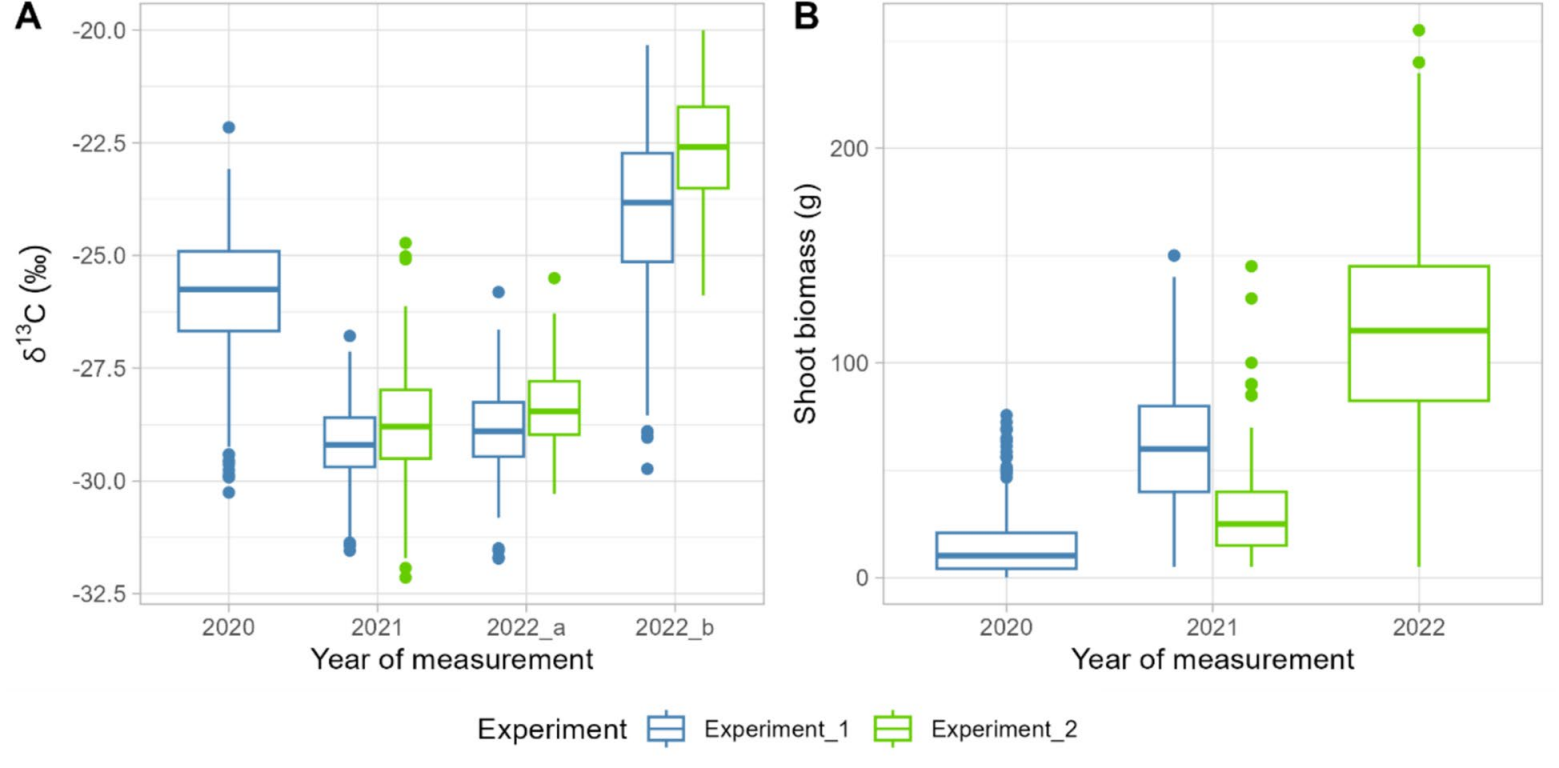
Phenotypic data variability for shoot traits in the *V. berlandieri* population. Boxplots of δ^13^C (**A**) measured in Experiment 1 (blue) and Experiment 2 (green). Boxes represent the interquartile range (25th – 75th percentiles), with the horizontal line indicating the median. Whiskers extend to the most extreme data point within 1.5 × the interquartile range. Values beyond this range are shown as individual outliers. The δ.^13^C has been measured in 2020 for Experiment 1, in 2021, and twice in 2022 for both Experiment 1 and Experiment 2. Boxplots of annual shoot biomass produced (**B**) measured in Experiment1 (blue) and Experiment 2 (green). The annual shoot biomass has been measured twice for each experiment: in 2020 and 2021 for Experiment 1, and in 2021 and 2022 for the Experiment 2

The water use efficiency of *V. berlandieri* genotypes was evaluated by δ^13^C measurements done in 2020, 2021, and twice in 2022 (2022a and 2022b). In Experiment 1, which consisted of 183 genotypes planted in field in 2020, the mean δ^13^C values were −25.9‰, −29.2‰, −28.9‰, and −24.1‰ in 2020, 2021, 2022a, and 2022b, respectively. In Experiment 2, which consisted of 142 genotypes planted in field in 2021, the mean δ^13^C values were −28.7‰, −28.4‰, −22.6‰ in 2021, 2022a and 2022b, respectively (Fig. 1). The pre-dawn leaf water potential reached −0.1 MPa in 2020, −0.1 MPa in 2021, −0.3 MPa in 2022a and −0.5 MPa in 2022b. We did not calculate the coefficient of variation (CV) for δ^13^C because it was expressed relatively to a standard, and therefore it was not meaningful to calculate the coefficient of variation for this trait [41]. However, H^2^ was moderate for δ^13^C, ranging from 0.34 to 0.46 over years. H^2^ was 0 in 2022b when the water deficit was the most intense (−0.5 MPa, Additional Table 3). Water potential ranged from −0.1 MPa (no water deficit) to −0.5 MPa (moderate to strong water deficit) [42].

### A moderate to high heritability for scion shoot biomass induced by the rootstock

**Fig. 2:**
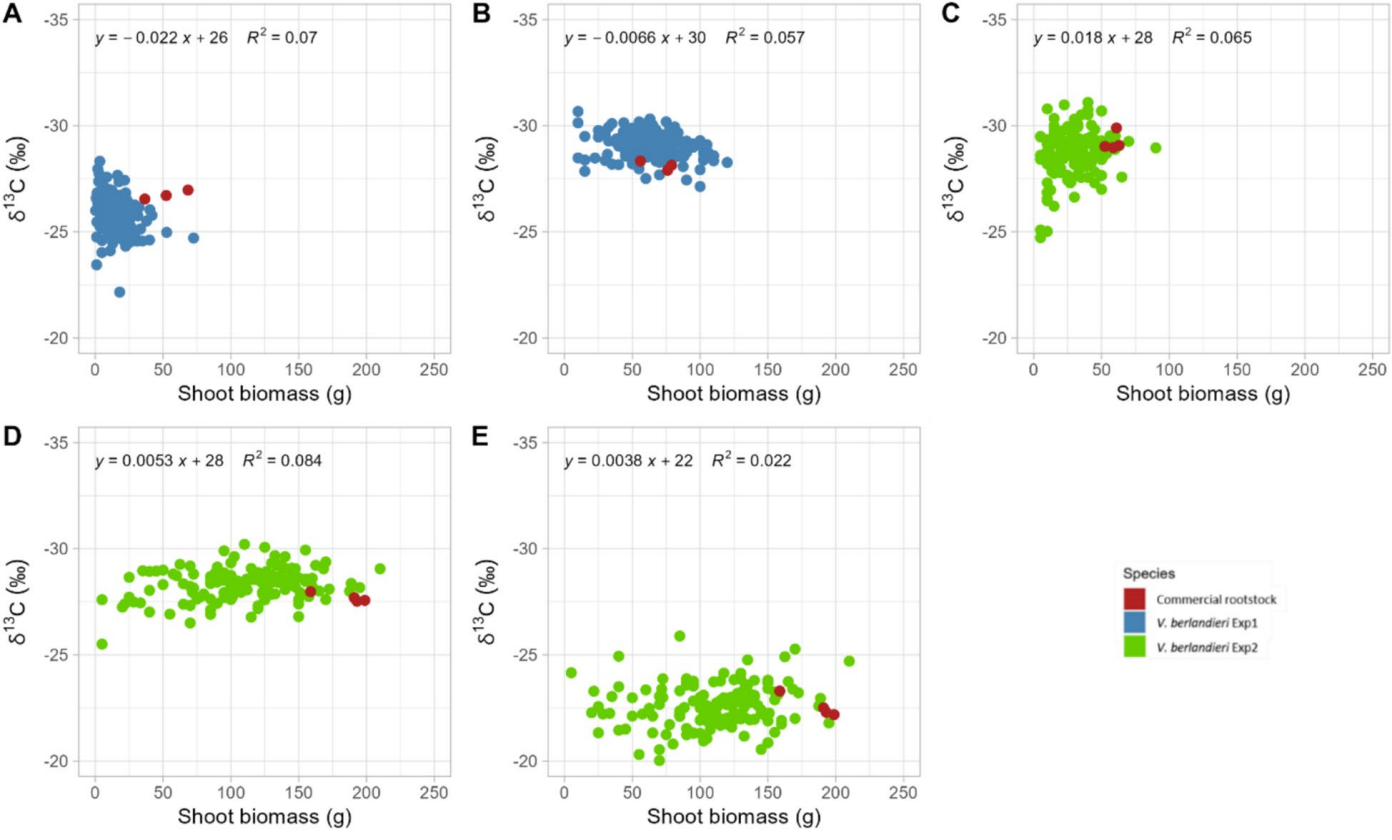
Correlation between shoot traits over years of measurements. Linear regression of the δ^13^C and the annual biomass measured for Experiment 1 in 2020 (**A**), 2021 (**B**), and for the Experiment 2 in 2021 (**C**), and 2022 (**D**, **E**). Regression equation is indicated on the top, R^2^ is the determining factor of correlation obtained with the Pearson method

During the experiments, annual shoot biomass ranged from 0.1 g to 150 g (Fig. 1). In Experiment 1, annual shoot biomass ranged from 0.1 g to 75.8 g (mean = 14.4 g) in 2020 and from 5.0 g to 150.0 g (mean = 63.7 g) in 2021. In Experiment 2, annual shoot biomass ranged from 5.0 g to 145.0 g (mean = 30.2 g) in 2021 and from 5.0 g to 255.0 g (mean = 113.9 g) in 2022. The CV for annual biomass in Experiment 1 ranged from 0.96 in 2020 to 0.46 in 2021 (Table 1). The CV for annual shoot biomass in Experiment 2 ranged from 0.70 in 2021 to 0.43 in 2022. A moderate-to-high H^2^ value was obtained over different years and experiments, ranging from 0.32 (Experiment 2, 2021) to 0.60 (Experiment 2, 2022). During the first years of growth and development addressed in this study, both in Experiments 1 and 2, there was no correlation between δ^13^C and annual shoot biomass (Fig. 2).

**Table 1:** Descriptive statistics of carbon isotope discrimination (δ^13^C) and the annual shoot biomass

Trait	Year	Exp 1						Exp 2					
		Max	Min	Mean	Stdev	H^2^	CV	Max	Min	Mean	Stdev	H^2^	CV
δ^13^C (‰)	2020	−22.2	−30.3	−25.9	1.3	0.34	-	NA	NA	NA	NA	NA	-
	2021	−26.8	−31.5	−29.2	0.8	0.36	-	−24.7	−32.1	−28.7	1.2	0.36	-
	2022a	−25.8	−31.7	−28.9	0.9	0.46	-	−25.5	−30.3	−28.4	0.8	0.46	-
	2022b	−20.3	−29.7	−24.1	1.7	0.00	-	−20.0	−25.9	−22.6	1.2	0.00	-
Biomass (g)	2020	75.8	0.1	14.4	13.8	0.57	0.96	NA	NA	NA	NA	NA	NA
	2021	150.0	5.0	63.7	29.1	0.34	0.46	145.0	5.0	30.2	21.0	0.32	0.70
	2022	NA	NA	NA	NA	NA	NA	255.0	5.0	113.9	48.6	0.60	0.43

The Experiment 1 (Exp 1) and the Experiment 2 (Exp2) are described separately. Stdev, H^2^, and CV correspond to the standard deviation, the broad sense heritability, and the coefficient of variation respectively. “- “ corresponds to data not collected

### Correlation of root-related and shoot traits

**Fig. 3:**
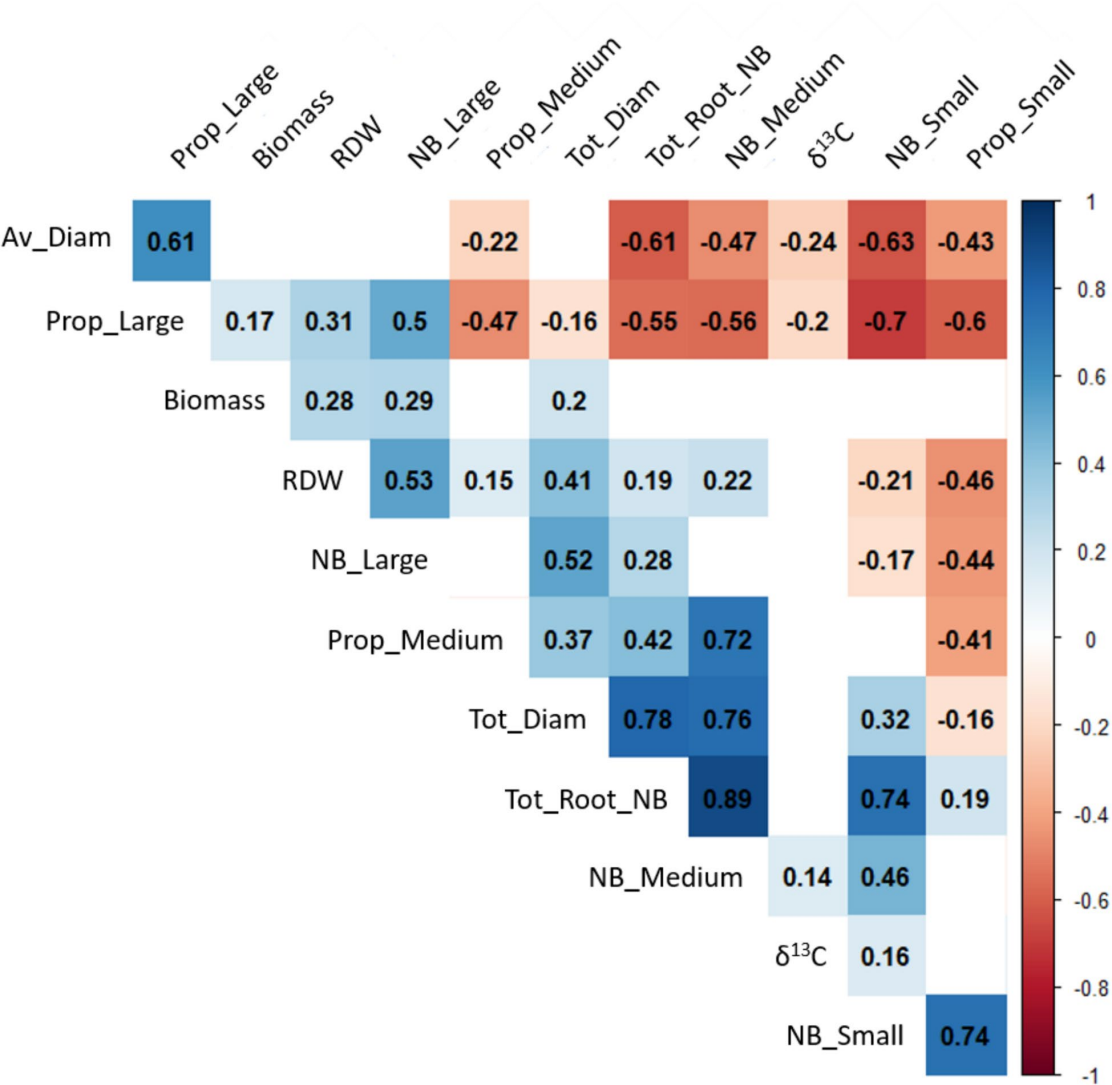
Correlation between above and below ground traits. Correlation matrix of genetic value (BLUEs obtained from a linear mixed model considering genotype and year as a fixed effect and blocks as random effects) of scion phenotype (δ.^13^C and annual biomass) and root related traits obtained from [37], including the total number of roots (Tot_Root_NB), the average root diameter (Av_Diam), the total root diameter (Diam_tot), the root dry weight (RDW), the number of small [0, 1) mm (NB_Small), medium [1, 2) mm (NB_Medium), and large [2, ∞) mm (NB_Large) as well as the proportion of each class of roots (Prop_Small, Prop_Medium, and Prop_Large respectively). Pearson correlation has been used, and only significant correlation have been labelled (*p*-value < 0.05)

Based on genetic values extracted from Eq. 4, the correlation between the root-related traits measured in a previous study [37] and aerial traits were stronger than that between different aerial traits (Fig. 3). The root traits considered were the average root diameter, the root dry weight, the total root diameter, the total root number and the number and proportion of small [0, 1) mm, medium [1; 2) mm, and large roots [2; ∞) mm.

The δ^13^C value was positively correlated with the number of medium roots (r = 0.14) and the number of small roots (r = 0.16). Annual biomass was positively correlated with root dry weight (r = 0.28), the number of large roots (r = 0.29), total root diameter (r = 0.20), and the proportion of large roots (r = 0.17). The δ^13^C value was negatively correlated with the average root diameter (r = −0.24) and the proportion of large roots (r = −0.2).

### GWAS for δ^13^C and shoot biomass revealed associated markers

**Fig. 4:**
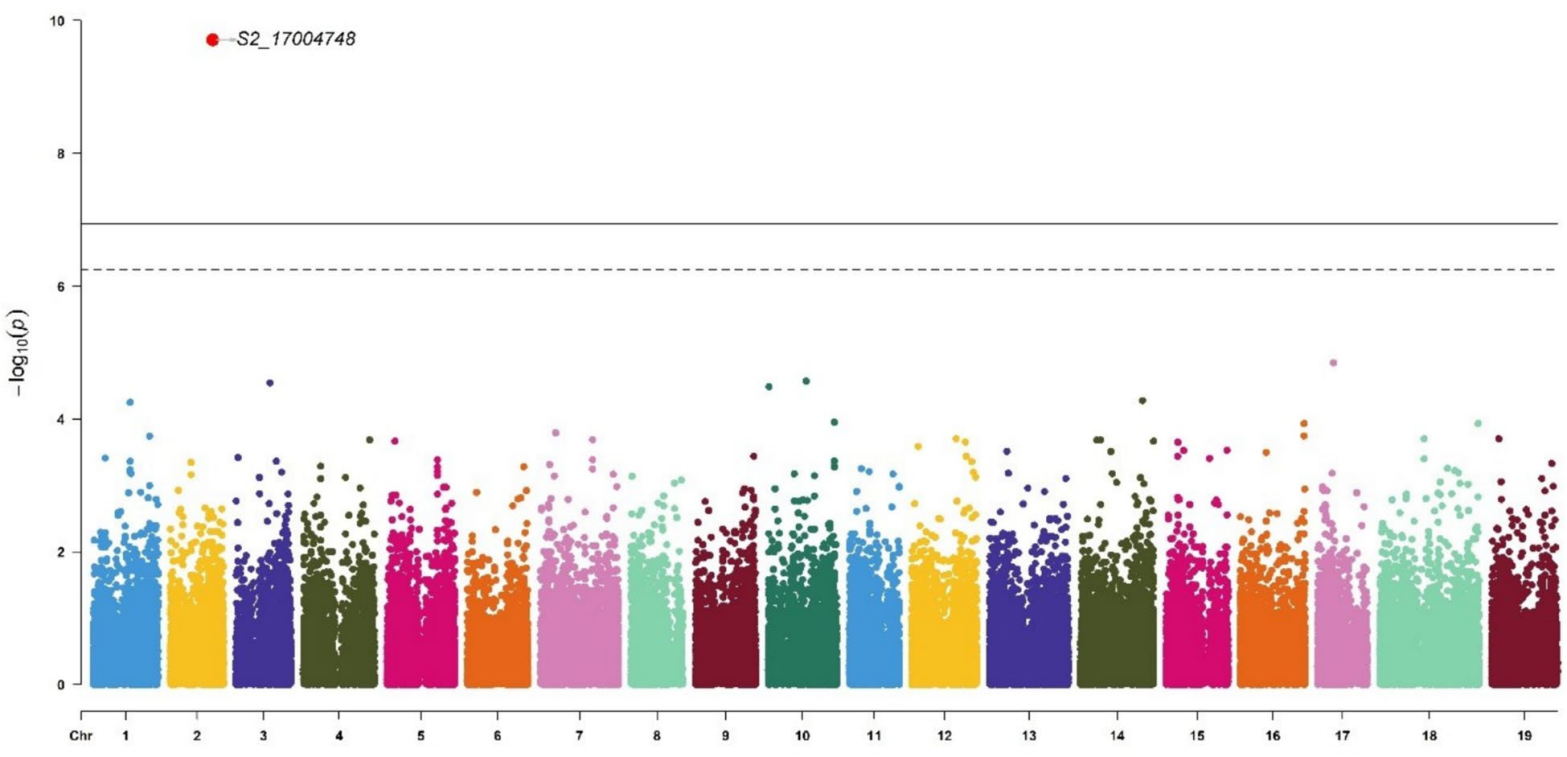
Manhattan plot of GWAS carried out on genetic values for δ^13^C. GWAS was done with BLINK. The lines correspond to significance thresholds based on Bonferroni correction (dotted α = 0.05 and full α = 0.01). Significant markers are indicated by large red points. The QQplot of the analysis is presented Additional Fig. 2a

**Fig. 5:**
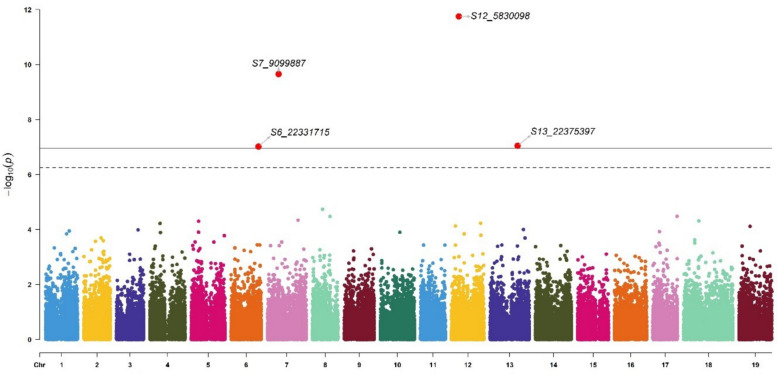
Manhattan plot of GWAS carried out on genetic values for the annual biomass. GWAS was done with BLINK. The lines correspond to significance thresholds based on Bonferroni correction (dotted α = 0.05 and full α = 0.01). Significant markers are indicated by large red points. The QQplot of the analysis is presented Additional Fig. 2b

We identified one marker significantly associated with δ^13^C (S2_17004748) (Fig. 4) and four associated with annual biomass (S6_22331715, S7_9099887, S12_5830098, and S13_22375397) (Fig. 5).

The marker associated with δ^13^C explained 54.4% of the genetic variance. This marker, located on chromosome 2, was linked to the Vitvi02g00466 gene, a homolog of AT4G22790.

The four markers associated with annual biomass explained from 7.9% to 10.7% of the genetic variance. These markers were located on chromosomes 6 (S6_22331715), 7 (S7_9099887), 12 (S12_5830098), and 13 (S13_22375397). The S6_22331715 marker was linked to the Vitvi06g00206 gene, which is homologous to AT5G19350. The S7_9099887 marker was linked to the Vitvi00g01403, Vitvi07g02508, and Vitvi07g02509 genes. The Vitvi07g02508 gene is homologous to AT4G37130. The S12_5830098 marker was linked to the Vitvi12g01915gene, which is homologous to AT1G22020. The S13_22375397 marker was linked to the Vitvi13g01237 gene, which is homologous to AT1G15780.

**Fig. 6:**
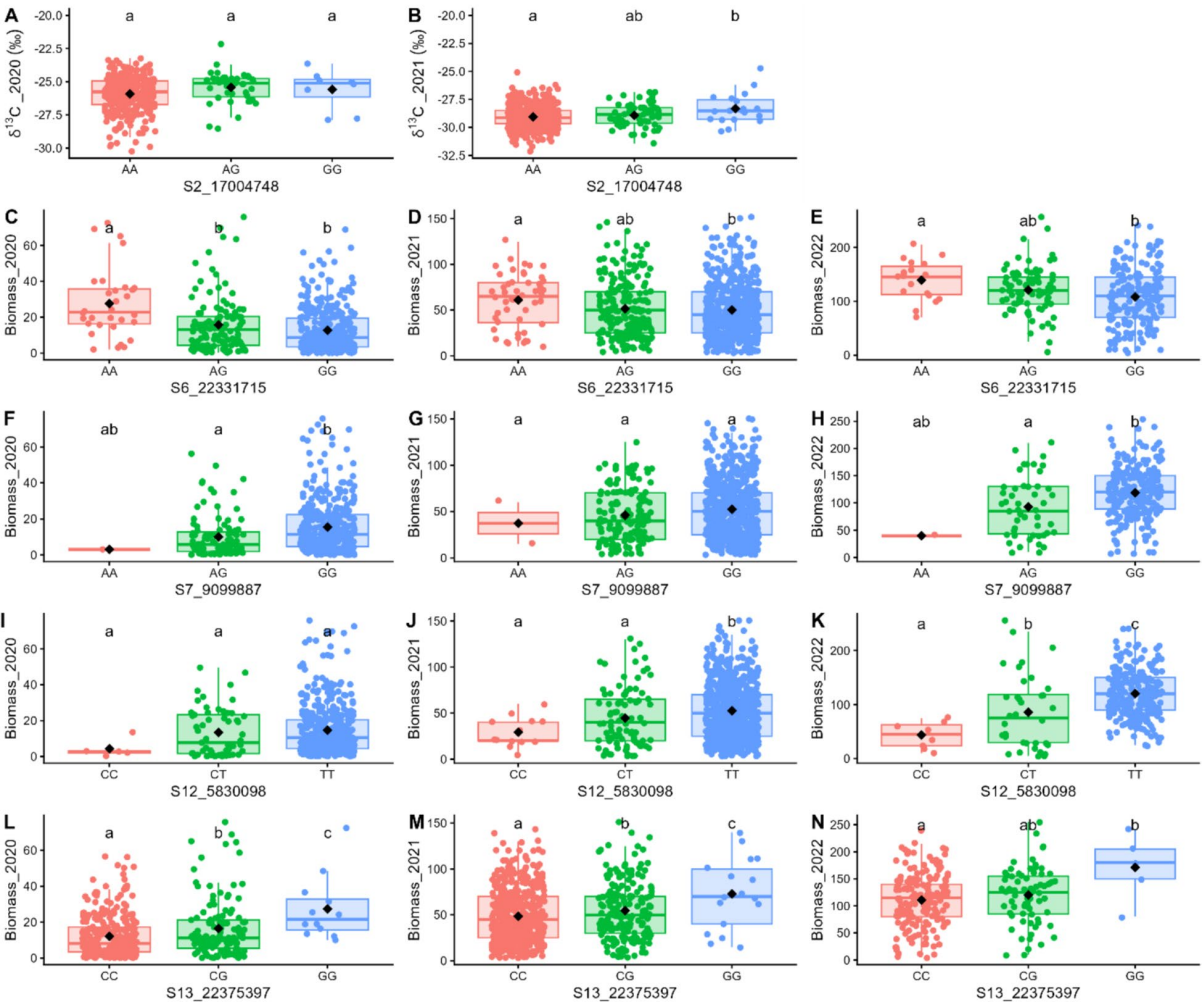
Effect of significant markers on raw phenotypic values. Results are indicated as boxplot of allelic groups for δ^13^C (‰) measured in 2020 (**A**) and 2021 (**B**) and for biomass (g per plant) measured in 2020 (**C**, **F**, **I**, **L**), 2021 (**D**, **G**, **J**, **M**), and 2022 (**E**, **H**, **K**, **N**). Boxes represent the interquartile range (25th – 75.^th^ percentiles), with the horizontal line indicating the median. Whiskers extend to the most extreme data point within 1.5 × the interquartile range. Statistically significant differences between groups were determined using Tukey’s test for multiple comparisons after ANOVA (*p* < 0.05)

A significant effect on raw phenotypic values was observed for all markers and all traits (Fig. 6). However, we did not observe the effect of every marker in all the years of measurement. Only the effects of S6_22331715 and S12_5830098, associated with the annual biomass, were observed in all years.

### Wild rootstocks outperformed commercial hybrids

The δ^13^C data for the commercial rootstocks followed a narrow distribution (Additional Fig. 3). During the most intense water deficit event (2022b), the commercial rootstocks had δ^13^C values in the middle of the distribution, from −26.8‰ to −22.5‰ in Experiment 1 and from −24.4‰ to −20.7‰ in Experiment 2. The range of values for *V. berlandieri* genotypes was larger, extending from −29.7‰ to −20.3‰ in Experiment 1 and from −25.5‰ to −20.0‰ in Experiment 2.

The distribution of biomass values for *V. berlandieri* genotypes covered a large range that increased over time (Additional Fig. 4). In both experiments, the commercial rootstocks performed very well during the first year of growth. However, a few *V. berlandieri* genotypes had performances at least as good as those of commercial rootstocks during the second and third years (data not shown for the third year).

## Discussion

Root traits influencing adaptation and productivity in perennial species are little studied due to the difficulty of accessing the belowground organ of a long-lived organism. Grafting offers a valuable approach to investigate root-mediated regulation of above-ground traits. In this study, a natural population of *V. berlandieri*, commonly used in grapevine rootstock breeding [29], was grafted onto a single *V. vinifera* cultivar (Riesling) and grown in a field trial conducted under the same site. This design enabled the characterization of genetic variability and architecture of root-regulated aerial traits in field conditions. We identified genetic variants associated with WUE and annual shoot biomass regulated by roots. These outcomes are promising not only for rootstock breeding of grafted crops but also to better understand which root genetic factors regulate aerial water use and growth.

### Genetic variability of water use efficiency regulated by roots

Plant water-use depends on water uptake by the roots and water loss through transpiration. Water uptake is subject to variability in architectural, morphological, anatomical, and physiological parameters affecting hydraulic properties [43]. Water loss is mainly controlled by stomatal opening, which is regulated principally by evaporative demand, together with osmotic, metabolic and hormonal signalling [44]. Thus, stomatal aperture have a major role in photosynthesis regulation impacting WUE, and δ^13^C [45].

In our study, the same scion genotype was used with each rootstock, avoiding the influence on genetic variation on genes expressed in leaves. Measurements were conducted in a field trial conducted under the same site, enabling correction for micro-environmental variation and allowing assessment of δ^13^C variability among plants. This demonstrated the influence of rootstock genotype on scion regulation and highlighted the critical role of the root system in regulating the soil–plant–atmosphere continuum. Although rootstock × scion interactions are well-documented [46, 47], they are rarely considered in quantitative genetic studies because of the extreme number of individuals needed.

Quantifying the amount of genetic variation in WUE regulated by roots is an essential question in rootstock breeding programs. Variability of WUE between grapevine cultivars has been demonstrated in previous studies [16, 48, 49]. Rootstock genotype has been shown to affect the δ^13^C measured on the scion in field conditions [50]. Moreover, the impact of the rootstock on scion WUE has already been highlighted, leading to the identification of QTL for water use efficiency regulated by the roots, but in a pot experiment based on a controlled water deficit [25]. In grapevine, variation between genotypes can be studied between varieties, between clones within varieties and between rootstock genotypes. Phenotypic variation for δ^13^C has been quantified at these three levels: 3‰ for varieties [48, 49], 3‰ for clones [50, 51], 3‰ for commercial rootstocks [50] to 6‰ for a rootstock pedigree population from an interspecific cross [25]. These results showed that not only the scion genotype but also the rootstock genotype play a role in determining water use efficiency of the plant.

In this field study, the plants experienced no water deficit during the two first seasons, but we were nevertheless able to observe δ^13^C variability in the scion in comparisons between rootstock genotypes. In the presence of a water deficit (2022a and 2022b), δ^13^C variation was greater within than between genotypes. The H^2^ was zero in 2022b, reflecting a strong impact of environment on the water use efficiency variability. The strong effect of the environment on the variation of δ^13^C was also highlighted in a study on eucalyptus at different seasons in field conditions [52]. In our study, in wet conditions, the H^2^ for δ^13^C was moderate (from 0.34 to 0.46), similar to that in *Pinus pinaster* (from 0.23 to 0.41, 39) and grapevine (from 0.33 to 0.65, 36). However, higher H^2^ has been reported for *Quercus robur,* ranging from 0.55 to 0.74 [54] and *Salix*, ranging from 0.66 to 0.77 [55], whereas a lower H^2^ value was obtained for a half-diallel maritime pine population (0.17), probably due to a combination of the measurement of δ^13^C on several wood rings and water stress events [56]. The sampling of wood rather than leaves results in a greater stability of the results due to the more integrative nature of wood measurements but it is less suitable for analyses involving shorter dry periods.

Even in the absence of a water deficit, δ^13^C remains a good indicator of plant WUE [57]. Elazab et.al. [57] studied four recombinant inbred lines of durum wheat in well-watered and water-deficient conditions. They observed correlations of δ^13^C measured on the leaf blade with root and aerial biomass accumulation in the well-watered context, whereas no clear pattern was observed in the presence of water deficit. On grapevine berry juice, it has been shown that δ^13^C measured in well-watered conditions can be used to discriminate the water-deficient responses of different cultivars [58]. Less consistent results were obtained when δ^13^C was measured in water-deficient conditions. A decrease in the H^2^ of δ^13^C during water-stress events in grasses [59] and wheat [60] had previously been reported, with the H^2^ decreasing from 0.57 in wet conditions to 0.12 during drought. This observation illustrates the genotype × environment interaction. When the plants are grown in non-limiting conditions, the environmental pressure is low, making it possible to observe the effects of rootstock genotype variability on phenotype. When environmental conditions are limiting, the impact of the environment increases, making it more difficult to observe the genetic effect (Additional Fig. 5). Moreover, under water-deficit conditions, plants progressively close their stomata. As the severity of the deficit increases, an increasing proportion of stomata become fully closed, thereby minimizing inter-genotypic variation until H^2^ ultimately approaches zero. Our results demonstrate that δ^13^C should be measured in non-limiting conditions for the comparison of rootstock genotype performances.

### Genetic variability of shoot biomass regulated by roots

The impact of roots in regulating shoot biomass growth has been well described for grapevine, where rootstock and the scion may affect many traits, including vegetative growth, yield and fruit quality [61]. Such effects have been also observed in other grafted crops [5] such as almond [62], or kiwifruit [63]. However, very little is known about the genetics underlying the regulation of aerial traits by roots. Our results confirmed the importance of rootstock influence on scion traits, even when using a single-species rootstock population (here *V. berlandieri)*, and gave further insights into the rootstock genetic regions controlling shoot biomass.

The shoot biomass varied considerably between *V. berlandieri* genotypes, with a moderate-to-high H^2^ (from 0.32 to 0.60). Bert et al. [64] previously reported a larger H^2^ range, from 0.26 to 0.86, for the “stem dry weight” of the scion grafted on a hybrid rootstock progeny between Cabernet-Sauvignon and a *Vitis* accession (Cabernet-Sauvignon × Riparia Gloire de Montpellier) in potted plants. The range of phenotypic values observed in our experiment is promising to allow selection of rootstock genotypes conferring low or high shoot biomass. Indeed, shoot biomass accumulation or vigor, as it is known in viticulture, may be an advantage or a disadvantage depending on edaphic and climatic conditions and the objectives of the producer in terms of yield and quality [65]. Moreover, shoot biomass is known to be positively correlated with vine fertility [15] and to affect the composition of grapes [14]. The genetic diversity of the *V. berlandieri* population could therefore be used to modulate the plant phenotypes, for its own root traits and shoot traits conferred to the scion, to promote grapevine adaptation to environmental conditions.

Shoot biomass accumulation and the WUE were not correlated. This finding is consistent with published results as no clear correlation has been reported between these two traits [52, 53, 56]. No common genetic marker was identified for growth and WUE, suggesting at least partial independent genetic determinism. Brendel et al. [56] highligthed phenotypic correlation between δ^13^C and ring width in maritime pine which was supposed to be related to environmental conditions. These observations revealed opportunities in breeding to select more water use efficient genotypes without impacting the shoot biomass of the plant in a context of no water limiting conditions.

### Link between root traits with water use efficiency and shoot biomass

In order to better understand the regulation of aerial phenotypes by roots we took advantage of a previously published work on the same rootstock population [37], where root-related traits were measured on plants cultivated in pots.

We found that δ^13^C was negatively correlated with the average root diameter and the proportion of large roots, and these two root traits were correlated with each other. Positive correlations were found between δ^13^C and the numbers of small and medium-sized roots. Genotypes with a high water use efficiency were characterized by a high number of fine roots. Root diameter has been shown to be associated with the root hydraulic conductivity, thick roots having a lower radial hydraulic conductivity and being more involved in axial water transport within the plant than in soil water extraction [66]. The individual root hydraulic properties directly impact the root system potential uptake and is proposed as an important proxy for drought adaptation of annuals [7] as well as perennials [43].

Root dry weight, the number (and proportion) of large roots and total root diameter were correlated with each other and all these traits were positively correlated with scion biomass reflecting the root:shoot ratio [4]. A link between root-related traits and aerial biomass accumulation has been reported in other ungrafted crops. For instance in wheat, vegetative dry weight is associated with the number of roots [67] and root dry weight [68].

In our study, we highlighted genetically linked root related traits that were correlated to water use efficiency and shoot biomass in a grafted field experiment. Selecting a rootstock ideotype for the adaptation of perennial crops is a complex question due to their long life cycles, which make perennial species are exposed to a broad range of biotic and abiotic environmental pressures [43]. Consequently, the perennial root-system ideotype must be adapted to diverse environmental conditions. The plant material used in this study was only two and three years old, corresponding to a juvenile stage. Further evaluation of the performance of these genotypes at adult stages should be done to test for the stability of the observed results along development. In perennials, the observation of the plant root system at mature stage represents a challenge due to its development during years. However, the juvenile stage during the first years of growth is a crucial step for plant establishment. Figuring out correlations between juvenile root-related and aerial agronomical traits in later stages could allow us to identify proxies as it has been shown in wheat, for which juvenile root-related traits were correlated with yield and plant height [69–71]. In grapevine, these traits could help to estimate aerial plant performances from juvenile rootstock rooting characteristics and may contribute to reduce the cost and duration of breeding efforts targeting root traits.

### Genetic architecture of water-use efficiency and shoot biomass regulated by roots

Most of the genes identified in association with WUE and aerial biomass are involved in stomatal regulation and plant development, which gives further support to their implication in the final expression of the measured phenotypes [25, 52, 72].

The S2_17004748 marker located on chromosome 2 explained 54.4% of the genetic variability for δ^13^C in the population. This result was highly surprising given the large number of factors affecting δ^13^C (transpiration rate, stomatal aperture, carbon assimilation, water-use efficiency, etc.). This marker was linked to a homolog of *AT4G22790*, which encodes a MATE family protein involved in stomatal aperture regulation in response to carbon dioxide concentration [73]. The *rhc1* mutant of *Arabidopsis*, in which the *AT4G22790* gene is altered, has a defective response to CO_2_. MATE-type proteins are transporters that connect increases in CO_2_ levels to the suppression of HT1, a protein kinase that inhibits the stomatal closure triggered by CO_2_. Our results are consistent with the function assigned to this gene, and the involvement of the root system in δ^13^C regulation further supports the plausibility of this finding. However, [74] studied stomatal signalling in *rhc1* mutants in conditions of high and low CO_2_ concentration and found no clear decrease in the sensitivity of stomatal closure to CO_2_. In previous GWAS for water deficit tolerance in grapevine grown in field, no marker was identified on chromosome 2 [26, 27]. In a quantitative genetic study of δ^13^C in grapevine grown in pots, Marguerit et al. [25] identified four QTL in a grafted progeny (*V. riparia* × Cabernet sauvignon) in a greenhouse experiment that did not colocalize with the markers identified here. The δ^13^C is linked to stomatal regulation which largely depend of evaporative demand. Since we observed a large contribution of environment to the phenotypic variance and because of the contrasted genetic material used in previous studies on grapevine, the identification of colocalized QTLs was unlikely. Indeed, unlike previous studies carried on grapevine, this study focused on a single-species population of *V. berlandieri*. This population was obtained by sampling in a dry chalky area of Texas. We expected an adaptation to water deficit of this species in this area, which may account for the detection of new genetic markers involved in plant water status responses. While the impact of rootstock genotype on the scion phenotype regulation has been observed in various grafted species, including grape [5], the identification of genetic variants at the rootstock level is rare. The identification of genetic markers from rootstock genotypes associated with the variability of the scion water responses revealed genetic-based regulation of this trait by the rootstock.

Four markers were associated with the plant annual shoot biomass trait. One of these markers, *Vitvi06g00206*, is homologous to *AT5G19350*, an RRM domain-containing protein that is very common and involved in various biological processes [75]. In *Arabidopsis thaliana*, the gene encoding the RBP45d protein has been implicated in the control of flowering time [76, 77]. Moreover, RBP45d has also recently been associated with vegetative growth and root development [78]. Loss-of-function mutations of the RBP45d gene led to a shorter primary root phenotype in mutants than in the wild type. This is meaningful in our experimental context of a grafted crop because the rootstock genotype is the source of vegetative trait variability. Decreases in root length affect the depth of the root system, limiting the water resources available to the plant. The *Vitvi07g02508* gene is homologous to *AT4G37130*, encoding NUP58 (nuclear pore complex protein) [79]. In *A. thaliana*, NUP58 is involved in the hormonal signalling pathways for auxins and gibberellins [80] and in plant growth and development, through the regulation of light perception, leaf organogenesis and flowering [80]. *Vitvi12g01915* is a homolog of *AT1G22020*, which functions as a serine hydroxymethyltransferase (SHM) in one-carbon folate metabolism and photorespiration [81]. *Vitvi13g01237*, a homolog of *AT1G15780*, is a mediator of RNA polymerase II transcription subunit 15a and is also known as Med15 [82]. Mediators such as Med15 are involved in the control of cell proliferation and flowering in response to light quality. Med15 has also been implicated in plant immunity and defence signalling [83, 84]. When considering the allelic distribution for the marker S13_22375397 linked to *Vitvi13g01237* in the population, the homozygous group “GG” was composed by genotypes issued from the sub-population 1 identified by [12]. This subpopulation evolved in a hot and wet area in which vegetative growth may not have been limited. Despite the short window explored due to rapid linkage disequilibrium decay, we were able to highlight the involvement of genes with meaningful functions for plant shoot biomass (hormonal regulation pathways, light responses, carbon metabolism, vegetative growth, and rooting ability). These markers accounted for 7.9% to 10.7% of the phenotypic variability. Because of the variability inherent to environmental effects and the juvenile stage of the plants analysed in this work, it is needed to validate these results at a mature stage before targeting them in breeding. In previous studies on grapevine, a QTL for shoot biomass was identified on chromosome 13 (VMC2C7) in the progeny of a *V. vinifera cv*. Cabernet-Sauvignon × *V. riparia cv*. Gloire du Montpellier cross [85]. One QTL was identified on the same chromosome by two statistical methods in a GWAS on 279 cultivars of *V. vinifera* [26]. However, no colocalization was observed between these QTL and the markers identified in our work. While these previous GWAS have been carried out for well-established mature plants, we worked on juvenile plants which were establishing in the field. Thus, the absence of colocalization can be due to the ontological difference between both studies. Moreover, Flutre et al. [26] highlighted QTLs that were not related to rootstock-scion interactions.

### Perspectives for breeding

Taking advantages of the research possibilities offered by grafting, we were able to detect phenotypic and genetic variability for root-mediated traits (WUE and shoot biomass) by controlling the genetic and environmental variability on genes expressed in aerial organs. To our knowledge, our study is the first GWAS on traits conferred by the rootstock on the scion performed with a natural population in field conditions. Our results contribute to the understanding of the mechanism through which roots regulate key aerial phenotypes, which is a fundamental question for the adaptation to the new biotic and abiotic constraints resulting principally from climate change. The capacity of the rootstock to maintain growth in the presence of water deficit is a crucial objective for breeding programs. Our results demonstrate that the genetic architectures of growth- and water-deficit response-related traits are independent, allowing selection without trade-offs for these traits, as already demonstrated for Populus [86], maritime pine [53], and Eucalyptus [52]. According to our observations, breeding strategies for water deficit tolerance would be limited to low and moderate water deficit conditions as long as heritability maintain moderate values. Increasing plants WUE might allow a lower water consumption during water deficit periods allowing to maintain edaphic water resources and delay strong water deficit symptoms. While we were unable to consider strong water deficit conditions in GWAS for young plants, we could expect lower susceptibility to water deficit in a mature context, when root system is deeper developed, making it possible to observe a genotype contribution to the δ^13^C phenotypic variance. Thus, breeding strategies could be relevant for drought resilience in broader environmental conditions.

In this study, we not only addressed a fundamental question regarding the identification of favourable alleles but also identified high performing genotypes that can be directly used as new rootstocks in vineyards and as potential parents in breeding programs. However, the mechanisms governing the rootstock influence on the scion water deficit still need to be deciphered. Future studies should also address key questions about the rootstock phenotypes with the greatest effect on fruit yield and quality under different scenarios of water limitation. Several rootstock-scion combinations should be tested as well in order to consider rootstock-scion interactions.

## Conclusions

This study highlights the genetic architecture of the impact of rootstocks on grapevine aerial traits, particularly water-use efficiency and biomass production, to improve drought resilience and support sustainable agricultural practices. We explored the genetic basis of water-use efficiency and shoot biomass regulated by roots in a natural population of *V. berlandieri* originated from an arid region. Taking advantage of the research possibilities offered by grafting, we were able to detect phenotypic and genetic variability for root-mediated traits (water-use efficiency and shoot biomass) by controlling the genetic and environmental variability on genes expressed in aerial organs. Our results provide evidence of the mechanisms through which roots regulate key aerial phenotypes for adaptation to the new biotic and abiotic constraints resulting principally from climate change. In addition, the results obtained are of potential value for breeding rootstocks at juvenile stage. Moreover, the highlighted genes represent a unique opportunity for accelerating plant improvement and adaptation with a focus on the roots system.

## Supplementary Information

Supplementary Material 1.

## Data Availability

Data for this study are available at: *V. berlandieri* reference genome: NCBI, BioProject ID: PRJNA886625. *V. berlandieri* population GBS data: NCBI, BioProject ID: PRJNA886619. Phenotypic data: Available on request to the corresponding author.

## Abbreviations

BIC: Best information criteria
CV: Coefficient of variation
BLINK: Bayesian information and linkage disequilibrium iteratively nested keyway
BLUE: Best linear unbiased estimate
BLUP: Best linear unbiased predictor
GAPIT: Genome association and prediction integrated tool
GWAS: Genome wide association study
H^2^: Broad sense heritability
HSD: Honestly significant difference
PW: Plant weight
QTL: Quantitative trait loci
SNP: Single nucleotide polymorphism
WUE: Water use efficiency
δ^13^C: Carbon isotope discrimination
Ψ_pd_: Pre-dawn water potential
