# Supplementary material for: Genome-wide association study of water-use efficiency and shoot biomass conferred by V. berlandieri rootstocks in grapevine
Source: BMC Plant Biol. 2026 Mar 27;26:805. doi: 10.1186/s12870-026-08542-6 (PMC13147898; doi:10.1186/s12870-026-08542-6)
Supplement: Supplementary file 1 — Supplementary Material 1. [file 12870_2026_8542_MOESM1_ESM.docx]

**Additional files**

Additional table 1: Blocks composition in the experimental plot

| **Year of planting** | **Number of genotypes** | **Number of plants** | **Block number** |
| --- | --- | --- | --- |
| 2020 | 183 | 183 | 1 |
| 2020 | 136 | 136 | 2 |
| 2020 | 97 | 97 | 3 |
| 2020 | 66 | 66 | 4 |
| 2020 | 37 | 37 | 5 |
| 2021 | 57 | 81 | 6 |
| 2021 | 87 | 223 | 7 |

Additional table 2: Weather monthly record in the field during experiments.

| **Year** | **Month** | **Rainfall (mm)** | **T_max (°C)** | **T_min (°C)** | **T_av (°C)** | **Radiation (daJ/cm²)** |
| --- | --- | --- | --- | --- | --- | --- |
| 2020 | Jan | 45 | 12.7 | 5.3 | 8.5 | 1552 |
| 2020 | Feb | 47.5 | 15.5 | 6.2 | 10.4 | 2478 |
| 2020 | Mar | 102.5 | 15.3 | 5.9 | 10.5 | 3902 |
| 2020 | Apr | 94 | 20.9 | 10.2 | 15.2 | 4903 |
| 2020 | May | 109.5 | 23.8 | 13 | 18.7 | 6998 |
| 2020 | Jun | 67.5 | 23.7 | 13.8 | 18.4 | 6590 |
| 2020 | Jul | 2.5 | 29.3 | 16.6 | 22.7 | 7748 |
| 2020 | Aug | 46.5 | 29.3 | 17.4 | 22.6 | 6183 |
| 2020 | Sep | 64.5 | 26.4 | 14 | 19.7 | 4822 |
| 2020 | Oct | 152 | 18.3 | 10 | 13.5 | 2749 |
| 2020 | Nov | 11.5 | 17.2 | 7.1 | 11.3 | 2051 |
| 2020 | Dec | 233 | 11.9 | 5.7 | 8.2 | 1118 |
| 2021 | Jan | 126.5 | 10.1 | 3.3 | 6.1 | 1533 |
| 2021 | Feb | 67 | 14.9 | 7.2 | 10.3 | 1966 |
| 2021 | Mar | 19 | 16.2 | 5.3 | 10.4 | 4373 |
| 2021 | Apr | 24.5 | 18.7 | 7.2 | 12.9 | 5848 |
| 2021 | May | 113.5 | 20 | 9.7 | 14.8 | 6241 |
| 2021 | Jun | 180 | 25.9 | 15.5 | 20.5 | 6678 |
| 2021 | Jul | 40.5 | 25.8 | 16.4 | 20.7 | 6275 |
| 2021 | Aug | 26.5 | 26.1 | 15.6 | 20.6 | 6060 |
| 2021 | Sep | 61.5 | 25.9 | 15 | 19.8 | 4417 |
| 2021 | Oct | 29.5 | 20.5 | 9.1 | 14 | 3535 |
| 2021 | Nov | 75 | 12.5 | 5.2 | 8.2 | 1749 |
| 2021 | Dec | 140.5 | 12.9 | 4.7 | 8.1 | 1436 |
| 2022 | Jan | 52.5 | 9.7 | 2.1 | 5.2 | 1706 |
| 2022 | Feb | 44 | 14.3 | 5.4 | 9.3 | 2471 |
| 2022 | Mar | 38 | 16.4 | 7.1 | 11.3 | 3788 |
| 2022 | Apr | 48.5 | 18.2 | 8.3 | 12.8 | 4992 |
| 2022 | May | 31.5 | 25.9 | 13.7 | 19.5 | 7479 |
| 2022 | Jun | 95 | 27.8 | 16.3 | 21.8 | 6860 |
| 2022 | Jul | 3 | 31.5 | 17.7 | 24.5 | 8409 |
| 2022 | Aug | 25.5 | 31.9 | 18.9 | 25 | 6581 |
| 2022 | Sep | 50.5 | 26 | 14.2 | 19.6 | 5019 |
| 2022 | Oct | 63.5 | 24 | 14.5 | 18.5 | 2840 |
| 2022 | Nov | 134.5 | 16.6 | 8.3 | 11.4 | 1851 |
| 2022 | Dec | 54.5 | 12.3 | 5.4 | 8.2 | 1369 |
| 2023 | Jan | 117.5 | 10.5 | 4.4 | 6.9 | 1357 |
| 2023 | Feb | 56 | 12.9 | 2.1 | 6.9 | 2744 |
| 2023 | Mar | 92.5 | 16.7 | 7.5 | 11.6 | 3610 |
| 2023 | Apr | 56.5 | 18.9 | 8.9 | 13.7 | 5572 |

Additional table 3: Pre-dawn water potential measured before leaves sampling. The water deficit correspondance is indicated according to (36). Two measrement ware carried out in 2022 corresponding to two water availability periods (2022a and 2022b).

| **Date** | **Year** | **ψ_pd_ (Mpa)** | **Water deficit correspondance** | **Number of plants** |
| --- | --- | --- | --- | --- |
| 11^th^ Sept | 2020 | -0.1 | Abs | 64 |
| 28^th^ Jul | 2021 | -0.1 | Abs | 51 |
| 03^rd^ Sept | 2021 | -0.1 | Abs | 51 |
| 28^th^ Jun | 2022 | -0.1 | Abs | 55 |
| 28^th^ Jul | 2022 | -0.3 | Moderate | 55 |
| 11^th^ Aug | 2022 | -0.5 | Moderate to strong | 18 |


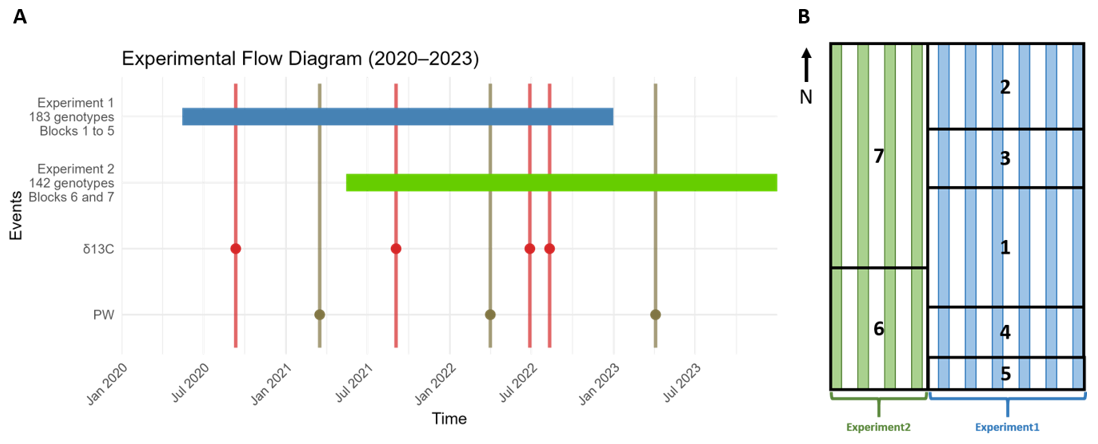


Additional fig. 1: Experimental procedure (A) over the three experimental years.

The field duration are indicated by colored bar (Experiment 1 in blue and Eperiment 2 in green). The δ^13^C and PW measurements are indicated by dots. Experimental design of the field plot (B). The Experiment1 is composed by 6 rows which were planted in 2020 within 5 blocks (blue) and the Experiment2 is composed by 4 rows which were planted in 2021 within 2 blocks (green).


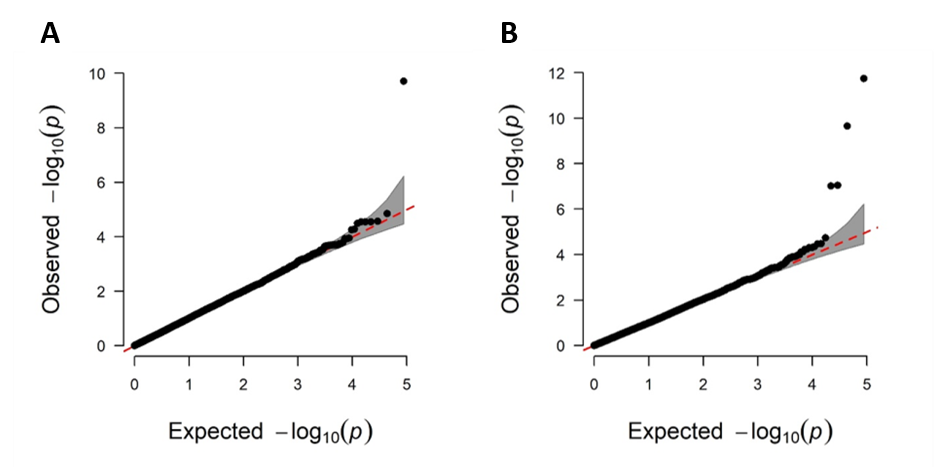


Additional fig. 2: QQ plots of GWAS carried out on genetic values for δ^13^C (a) and annual shoot biomass (b).


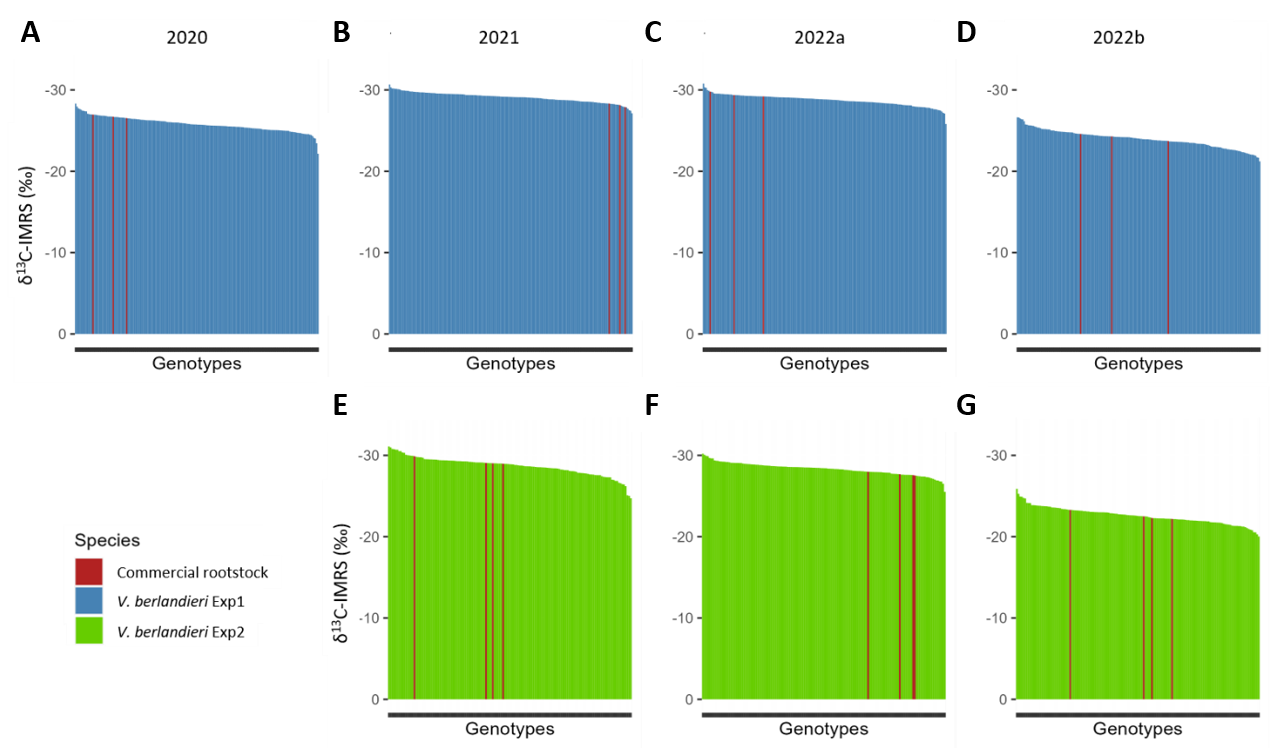


Additional fig. 3 : Distributions of the δ^13^C phenotypic values over years.

Measured for the Experiment1 measured in 2020 (a), 2021 (b), and 2022 (c, d) and Experiment 2 measured in 2021 (e) and 2021 (f, g). Commercial rootstocks (Börner, 110R, SO4, and 5BB) position are indicated with red lines.


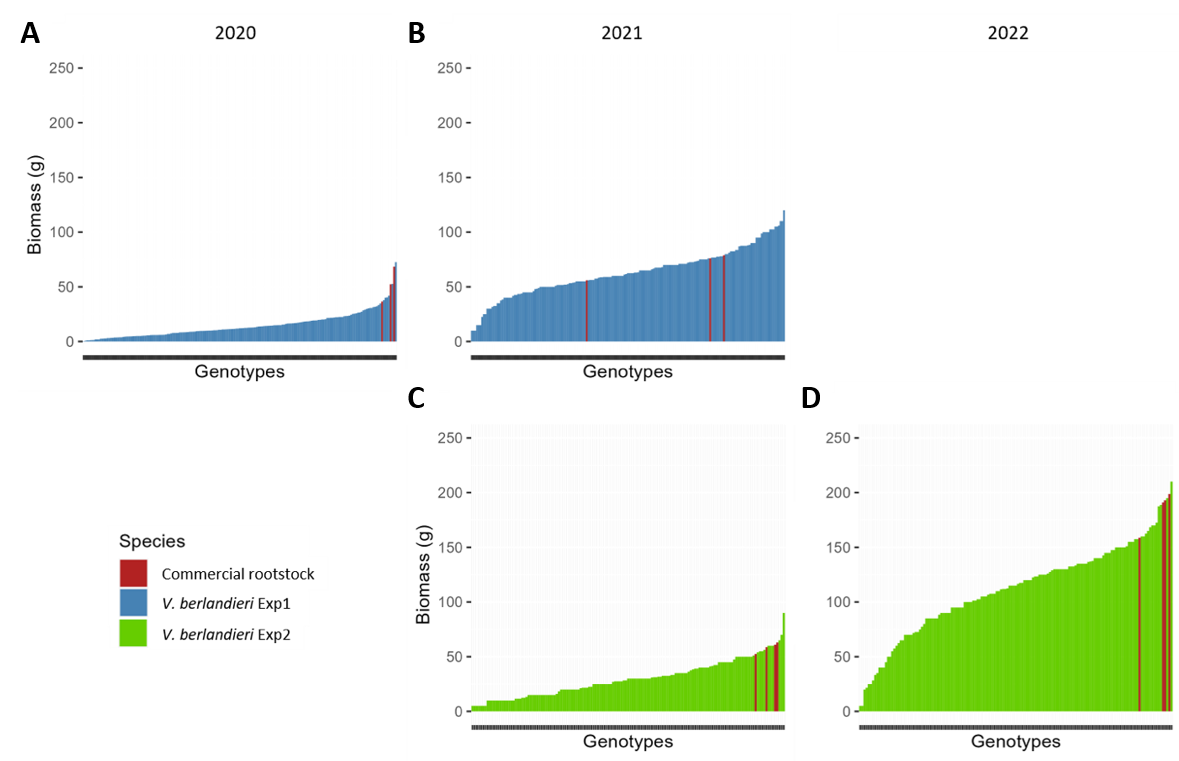


Additional fig. 4 : Distributions of the annual biomass phenoytpic values over years.

Measured for the Experiment1 measured in 2020 (a), 2021 (b), and 2022 (c) and Experiment 2 measured in 2021 (d) and 2022 (e). Commercial rootstocks (Börner, 110R, SO4, and 5BB) position are indicated with red lines.


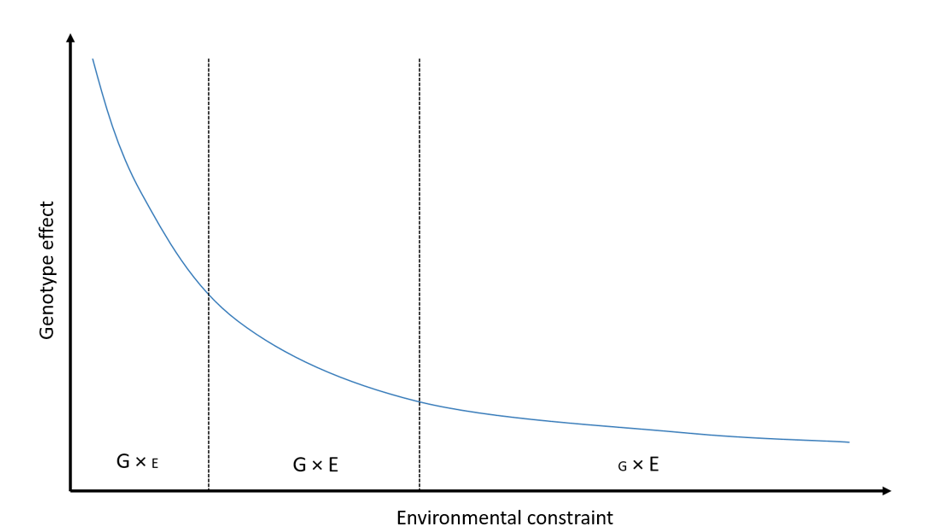


Additional fig. 5 : Theorethical impact of environmental constraint on the genotype variability expression on phenotype.

In the first section of the curve, the environnemental constraint is low, thus the phenotype variability is mainly linked to the genetic variability. In the second section of the graphic, the environmental constraint is moderate, thus, the observable phenotype results from the interaction of the genotype and the phenotype. In the third section, the environmental constraint is high, which reduces the expression of the genoytpe variability and increases the environmental part of the phenotype variability.
